# Coordinated kinetics of humoral, T-cell, and cytokine responses to SARS-COV-2 in West African healthcare workers: a multicenter longitudinal study in university hospitals of Abidjan, Côte d’Ivoire

**DOI:** 10.3389/fimmu.2025.1669517

**Published:** 2025-11-21

**Authors:** Amah Patricia Victorine Goran-Kouacou, Oppong Richard Yéboah, Yida Jocelyne Séri, Aya Ursule Aniela Assi, Adjoumanvoulé Honoré Adou, Salimata Moussa, Brou Doris Oura, Koffi N’Guessan, Kouabla Liliane Siransy, Séry Romuald Dassé

**Affiliations:** Department of Immunology and Allergology, Faculty of Medicine, Félix Houphouët-Boigny University, Abidjan, Côte d’Ivoire

**Keywords:** SARS-CoV-2, COVID-19, healthcare workers, humoral immunity, T-cell response, cytokines, neutralizing antibodies, sub-Saharan Africa

## Abstract

**Background:**

Healthcare workers in resource-limited settings are frequently exposed to SARS-CoV-2, often with incomplete vaccine coverage. Yet, their adaptive immune responses remain poorly characterized in sub-Saharan Africa.

**Methods:**

We conducted a multicenter study involving 36 healthcare workers with RT-PCR-confirmed SARS-CoV-2 infection (83.3% vaccinated) and 40 strictly unvaccinated, COVID-19-naïve controls from three university hospitals in Abidjan. Blood samples were collected on Days 0, 7, 14, 21, and 28. IgM and IgG were measured by ELFA (Mini VIDAS^®^), neutralizing antibodies with CHORUS TRIO^®^, lymphocyte subsets by flow cytometry, and Th1/Th2/Th17 cytokines using bead-based multiplex assays.

**Results:**

Infected participants showed strong antibody production at baseline. IgG levels were closely linked to neutralizing activity (ρ = 0.83; *p < 0.0001*), and CD4^+^ T-cell counts correlated with IL-2 (ρ = 0.71; *p < 0.0001*). We observed early activation across Th1 (IFN-γ, TNF-α), Th2 (IL-4, IL-10), and Th17 (IL-17A) pathways. IL-17A levels were higher in asymptomatic individuals (*p = 0.031*). Over time, IgM and pro-inflammatory cytokines declined, while IgG remained stable and regulatory cytokines rose.

**Conclusion:**

This cohort developed a broad immune response involving antibodies, T cells, and cytokines. The IL-17A pattern seen in asymptomatic cases may reflect effective mucosal control. These findings contribute essential data from a region where immune profiling remains limited.

## 1 Introduction

Since the emergence of SARS-CoV-2, healthcare workers (HCWs) have remained at high risk of infection, particularly in resource-limited settings where vaccine coverage is often incomplete (1, 2). Their repeated exposure makes them a key population for studying adaptive immune responses. Protective immunity against SARS-CoV-2 involves both humoral and cellular arms. Virus-specific antibodies, especially against the spike protein, contribute to neutralization (3), but their levels tend to decline over time (4). In parallel, CD4^+^ and CD8^+^ T-cell responses play essential roles in viral clearance and long-term protection (2, 5, 6). These responses are regulated by a complex cytokine environment that reflects T-helper polarization, including Th1 (e.g., IFN-γ, TNF-α), Th2 (e.g., IL-4, IL-10), and Th17 (e.g., IL-17A) pathways (7, 8). IL-17A, in particular, has been implicated in mucosal defense and may limit viral replication without systemic inflammation (9, 10). This cytokine pattern is especially relevant in asymptomatic cases (11, 12). In sub-Saharan Africa, immune dynamics may be shaped by region-specific factors such as exposure to diverse pathogens, chronic parasitic infections, or prior contact with endemic human coronaviruses (7). Yet despite these unique features, immunological studies in African populations remain scarce (13). To address this gap, we investigated post-infectious adaptive immunity in HCWs from Côte d’Ivoire, a population exposed to high SARS-CoV-2 circulation and heterogeneous vaccine access. We specifically analyzed the kinetics and coordination of virus-specific antibodies, lymphocyte subsets, and cytokine profiles, seeking to identify immune signatures associated with clinical presentation.

## 2 Materials and methods

### 2.1 Study design, setting, and participants

This investigation was conducted as part of a multicenter project on SARS-CoV-2 immune responses among healthcare workers in Côte d’Ivoire. The study was observational, descriptive, and longitudinal, carried out between January 2022 and June 2023 in three tertiary hospitals of Abidjan: the University Hospitals of Cocody, Treichville, and Angré. These facilities were selected for their high patient turnover and their diverse range of healthcare services, providing heterogeneous exposure conditions for healthcare staff.

The study population included medical, paramedical, and administrative personnel aged 18 years or older who provided written informed consent. Participants were stratified according to occupational exposure to SARS-CoV-2: low risk (administrative staff with no direct patient contact), intermediate risk (staff working with patients of unknown or suspected COVID-19 status), and high risk (staff with regular contact with confirmed COVID-19 cases, particularly in emergency and intensive care units).

A total of 275 participants were enrolled, including 36 SARS-CoV-2 RT-qPCR-positive cases and 40 uninfected controls followed longitudinally. All controls were unvaccinated and tested negative for SARS-CoV-2 by RT-qPCR at inclusion. Although asymptomatic infections could not be completely excluded, seronegative screening minimized potential misclassification bias. Although the cohort size was limited by logistical and financial constraints, stratification and random selection minimized recruitment bias. The modest number of confirmed cases was recognized as a potential limitation, possibly affecting statistical power and representativeness, and was explicitly considered when interpreting results.

### 2.2 Sampling and data collection

Baseline sampling (Day 0) consisted of venous blood collection (5 mL on EDTA and 5 mL on a dry tube) and nasopharyngeal swabbing. All samples were processed within two hours after collection. Serum and plasma were aliquoted to avoid repeated freeze-thaw cycles and stored at -80 °C until analysis.

Longitudinal follow-up was performed at Days 7, 14, 21, and 28 for RT-qPCR-positive participants and matched controls, allowing parallel evaluation of humoral, cellular, and cytokine dynamics. A standardized clinical and exposure questionnaire was administered at inclusion to capture sociodemographic characteristics, comorbidities, vaccination status, and occupational risk level.

### 2.3 Laboratory analyses

#### 2.3.1 Viral RNA detection

Nasopharyngeal swabs placed in viral transport medium were analyzed for SARS-CoV-2 RNA using the KingFisher™ Duo Prime system (Thermo Fisher Scientific, Waltham, MA, USA) with the MagMAX™ Viral/Pathogen kit for automated extraction. Amplification was performed on a CFX96™ Real-Time PCR Detection System (Bio-Rad, Hercules, CA, USA) targeting the nucleocapsid (N) and RNA-dependent RNA polymerase (RdRp) genes. A cycle threshold (Ct) < 35 defined positivity. Each run included an internal control (cellular RNA), a certified positive control, and a negative control. Cycle threshold values were used qualitatively for case definition and not for viral load quantification.

#### 2.3.2 Serological assays (IgM and IgG anti-RBD)

Detection of antibodies directed against the receptor-binding domain (RBD) of the SARS-CoV-2 Spike protein was performed on the Mini-VIDAS^®^ analyzer (bioMérieux SA, Marcy-l’Étoile, France; Serial No. IVD7006414, Ref. 410417) using the VIDAS^®^ SARS-CoV-2 IgM and VIDAS^®^ SARS-CoV-2 IgG II kits. The assays rely on enzyme-linked fluorescent assay (ELFA) technology providing qualitative and semi-quantitative detection of specific antibodies.

IgM and IgG results were first obtained as index values (ratio of the sample Relative Fluorescence Value [RFV] to that of the calibrator), with an index ≥ 1.0 considered positive. For quantitative comparison, index values were converted to Binding Antibody Units per milliliter (BAU/mL) using the manufacturer-derived conversion factor 1 index = 20.33 BAU/mL, standardized to the 1st WHO International Standard (20/136). Accordingly, antibody titers ≥ 20 BAU/mL indicated seropositivity, and ≥ 250 BAU/mL represented a strong antibody response.

#### 2.3.3 Neutralizing antibody assay

Neutralizing antibodies against the S1 subunit of the Spike protein were quantified using the CHORUS TRIO^®^ semi-automated immunoanalytical system (Diesse Diagnostica Senese S.p.A., Siena, Italy; Serial No. 4341, P/N 81200) and the CHORUS SARS-CoV-2 “Neutralizing” Ab kit. The method is a competitive enzyme immunoassay in which antibodies in the sample compete with a labeled tracer for binding to the viral RBD/ACE2 complex. The degree of inhibition reflects neutralizing activity and is expressed in BAU/mL relative to the WHO International Standard (20/136). Results were classified as negative (< 20 BAU/mL), equivocal (20-49.9 BAU/mL), or positive (≥ 50 BAU/mL). Each analytical series included a stored master calibration curve, a certified positive control, and an internal negative control to ensure traceability and inter-assay reproducibility.

#### 2.3.4 T-cell immunophenotyping

T-lymphocyte subsets were quantified on a BD FACSCanto™ II flow cytometer (BD Biosciences, San Jose, CA, USA; Serial No. V3389002039) equipped with 488 nm and 633 nm lasers. Whole blood (50 µL, EDTA-treated) was stained with monoclonal antibodies anti-CD3 (UCHT1-FITC, Ref. 555332), anti-CD4 (RPA-T4-APC, Ref. 555349), anti-CD8 (RPA-T8-PE, Ref. 555635), and anti-CD45 (2D1-PerCP-Cy5.5, Ref. 564105). After a 20-min incubation at room temperature, erythrocytes were lysed with BD FACS™ Lysing Solution and washed in PBS. Acquisition included ≥ 10,000 lymphocyte-gated events. Data were analyzed using FACSDiva™ v8.0 software (BD Biosciences). Absolute lymphocyte subset counts were derived by combining flow cytometry percentages with total lymphocyte counts obtained from concomitant full blood counts and expressed as cells/µL.

#### 2.3.5 Cytokine profiling

Cytokines were quantified in serum using the BD™ CBA Human Th1/Th2/Th17 Cytokine Kit (Ref. 560484, BD Biosciences). The assay allows multiplex quantification of IL-2, IL-4, IL-6, IL-10, TNF-α, IFN-γ, and IL-17A by flow cytometry. Serum samples were centrifuged at 3,500 rpm for 5 min, heat-inactivated at 56 °C for 30 min, and diluted 1:4 before analysis. Fifty microliters of cytokine-capture beads were incubated with standards or samples and PE-labeled detection reagent for 3 h in the dark. Data were acquired on the BD FACSCanto™ II and analyzed using FCAP Array™ v3.0 software. Detection ranges (manufacturer data, 2022) were IL-2 (2-5,000 pg/mL), IL-4 (2-5,000), IL-6 (2.5-5,000), IL-10 (2.7-2,000), TNF-α (3.7-2,000), IFN-γ (3-5,000), and IL-17A (2-5,000 pg/mL).

### 2.4 Variables and data management

Variables included demographic characteristics, comorbidities, vaccination status, and occupational exposure level. Biological parameters included IgM and IgG anti-RBD, neutralizing antibodies, lymphocyte subsets, and cytokine concentrations. Data were double-entered into Microsoft Excel 2016, cross-checked for inconsistencies, and exported to SPSS v26.0 for analysis. All records were pseudonymized, and quality control ensured internal validity before database locking.

### 2.5 Statistical analysis

Data were analyzed using SPSS v26.0 (IBM Corp., Armonk, NY, USA). Quantitative variables were expressed as mean ± standard deviation (SD) or median (interquartile range, IQR), depending on distribution. Categorical variables were expressed as frequencies and percentages. Correlations between antibody titers (IgG, NAbs) and cytokine levels (IL-2, IFN-γ, TNF-α) were assessed using Spearman’s rank correlation coefficient (ρ). Group comparisons were performed using the Mann-Whitney U test for continuous variables and the Chi-square or Fisher’s exact test for categorical variables. Statistical significance was set at p < 0.05.

### 2.6 Ethical considerations

The study protocol was approved by the National Ethics Committee for Life Sciences and Health (CNESVS) of Côte d’Ivoire (Ref. 007-22/MSHP/CMU/CNESVS-km). All participants provided written informed consent prior to enrollment, and all procedures complied with the Declaration of Helsinki (2013) and national regulations on biomedical research ethics.

## 3 Results

### 3.1 Baseline characteristics of participants

Participant demographics and occupational data are summarized in **Table 1**. Cases and controls were comparable in median age (39.0 vs. 37.0 years), female proportion (63.9% vs. 67.5%), and median BMI (24.7 vs. 25.9 kg/m²). In contrast, hospital affiliation and occupational exposure level differed, with a higher proportion of high-risk exposure among cases (36.1% vs. 12.5%). All controls were unvaccinated, while 83.3% of cases had received at least one dose of a SARS-CoV-2 vaccine.

**Table 1:** Characteristics of participants.

Variable	Category	Cases (n = 36)	Controls (n = 40)
Sex	Female	23 (63.9%)	27 (67.5%)
	Male	13 (36.1%)	13 (32.5%)
Age (years)	Mean ± SD	40.7 ± 12.8	37.6 ± 9.1
	Median	39.0	37.0
BMI (kg/m²)	Mean ± SD	26.0 ± 4.3	25.8 ± 3.8
	Median	24.7	25.9
Professional category	Physician	10 (27.8%)	11 (27.5%)
	Registered nurse	11 (30.6%)	6 (15.0%)
	Nursing assistant	6 (16.7%)	5 (12.5%)
	Lab technician	2 (5.6%)	5 (12.5%)
	Administrative staff	4 (11.1%)	9 (22.5%)
	Other	3 (8.3%)	4 (10.0%)
Affiliated teaching hospital	Cocody	18 (50.0%)	32 (80.0%)
	Angré	11 (30.6%)	3 (7.5%)
	Treichville	7 (19.4%)	5 (12.5%)
Occupational exposure level	Low	6 (16.7%)	10 (25.0%)
	Intermediate	17 (47.2%)	25 (62.5%)
	High	13 (36.1%)	5 (12.5%)
COVID-19 vaccination status	Vaccinated	30 (83.3%)	0 (0.0%)
	Not vaccinated	6 (16.7%)	40 (100.0%)
Number of doses	Mean ± SD	1.5 ± 0.8	–
	Median	2	–

### 3.2 Immune profiles at inclusion: cases vs. controls

At Day 0, cases had significantly higher levels of IgM, IgG, and neutralizing antibodies than controls (36.4 vs. 4.3 BAU/mL, 556.3 vs. 272.2 BAU/mL, and 1469.6 vs. 921.8 BAU/mL, respectively; *p < 0.0001* for IgM and IgG, *p = 0.0017* for NAbs; **Table 2**). CD4^+^ and total CD3^+^ T cell counts were also higher in cases (2368.4 vs. 1560.8 cells/µL and 3525.4 vs. 2949.0 cells/µL; *p = 0.0054* and *p = 0.0463*), with no significant difference for CD8^+^ (*p = 0.2418*). Cytokine concentrations of IL-10, IFN-γ, TNF-α, IL-2, IL-4, and IL-17A were significantly higher in cases (*p < 0.01* for all), while IL-6 showed no significant difference (14.9 vs. 18.8 pg/mL; *p = 0.1988*; **Table 2**).

**Table 2:** Comparison of immune markers at day 0 between cases and controls.

Marker	Cases (median [IQR])	Controls (median [IQR])	P-value
IgM (BAU/mL)	36.4 [22.8-79.3]	4.3 [2.3-6.8]	< 0.0001
IgG (BAU/mL)	556.3 [458.7-662.3]	272.2 [213.2-326.1]	< 0.0001
NAbs (BAU/mL)	1469.6 [788.8-1547.2]	921.8 [388.4-1401.1]	0.0017
CD3 (cells/µL)	3525.4 [3146.3-4143.5]	2949.0 [2493.2-3745.6]	0.0463
CD4 (cells/µL)	2368.4 [2007.2-2502.6]	1560.8 [1192.1-1935.7]	0.0054
CD8 (cells/µL)	1060.8 [1004.6-1642.9]	1315.2 [1025.0-1638.0]	0.2418
IL-6 (pg/mL)	14.9 [13.7-17.7]	18.8 [10.7–21.3]	0.1988
IL-10 (pg/mL)	4.4 [3.7-5.0]	1.5 [1.4-1.6]	< 0.0001
IFN-γ (pg/mL)	0.4 [0.2-1.3]	0.3 [0.2-0.3]	0.0023
TNF-α (pg/mL)	2.9 [1.6-5.8]	1.2 [0.5-1.8]	< 0.0001
IL-2 (pg/mL)	2.5 [2.1-2.6]	0.2 [0.1-0.4]	< 0.0001
IL-4 (pg/mL)	2.1 [1.1-3.2]	0.1 [0.0-0.1]	< 0.0001
IL-17A (pg/mL)	13.5 [12.6-14.2]	4.7 [2.3-5.8]	< 0.0001

### 3.3 Comparison by symptom status at inclusion

Among infected individuals, most immune markers showed no significant differences between symptomatic and asymptomatic subgroups. No statistical differences were observed for IgM, IgG, NAbs, CD4^+^, CD8^+^, IL-2, IL-4, IL-6, IL-10, IFN-γ, or TNF-α. Only IL-17A was significantly higher in asymptomatic individuals (13.8 vs. 12.9 pg/mL; *p = 0.0311*; **Table 3**).

**Table 3:** Comparison of immune and cytokine markers at day 0 between symptomatic and asymptomatic cases.

Marker	Symptomatic (median [IQR])	Asymptomatic (median [IQR])	P-value
IgM	33.2 [21.9-73.4]	56.0 [22.8-87.0]	0.3258
IgG	551.4 [451.2-655.3]	586.8 [520.2-684.8]	0.5362
NAbs	1469.6 [505.3-1508.8]	1531.3 [1312.3-1700.3]	0.1344
CD3	3440.6 [1910.4-3959.7]	3901.7 [3432.9-4482.7]	0.0733
CD4	2395.9 [841.5-2877.6]	2329.5 [2301.7-2441.3]	0.9874
CD8	1065.2 [1003.0-1081.7]	1056.4 [1006.1-2181.1]	0.3834
IL-2	2.5 [1.1-2.9]	2.4 [2.4-2.5]	0.9873
IL-4	1.7 [0.95-3.10]	2.3 [1.8-3.3]	0.3028
IL-6	15.1 [13.5-16.7]	14.9 [14.2-18.0]	0.5262
IL-10	4.8 [3.9-5.1]	4.3 [3.7-4.8]	0.0817
IFN-γ	0.38 [0.38-2.07]	0.38 [0.17-0.54]	0.1456
TNF-α	3.0 [1.8-5.8]	2.9 [1.7-5.8]	0.7634
IL-17A	12.9 [12.5-13.7]	13.8 [13.3-14.9]	0.0311

### 3.4 Immune correlations at inclusion in cases

A strong positive correlation was observed between IgG and neutralizing antibodies (ρ = 0.83; *p < 0.0001*). Other significant correlations included CD4^+^ with IL-2 (ρ = 0.71; *p < 0.0001*), CD4^+^ with IL-10 (ρ = 0.46; *p = 0.005*), and CD4^+^ with TNF-α (ρ = 0.41; *p = 0.013*). IL-6 also correlated with both IL-10 and TNF-α, and a strong correlation was noted between IL-2 and IL-4 (ρ = 0.62; *p < 0.0001*; **Table 4**).

**Table 4:** Correlations between immune markers in positive cases at day 0.

Correlated markers	Spearman’s ρ coefficient	P-value
IgG ↔ NAbs	0.83	< 0.0001
CD4 ↔ IL-2	0.71	< 0.0001
CD4 ↔ IL-10	0.46	0.005
CD4 ↔ TNF-α	0.41	0.013
IL-6 ↔ TNF-α	0.68	< 0.0001
IL-6 ↔ IL-10	0.57	< 0.0001
IL-2 ↔ IL-4	0.62	< 0.0001

### 3.5 Longitudinal evolution of immune markers in cases

**Figure 1:**
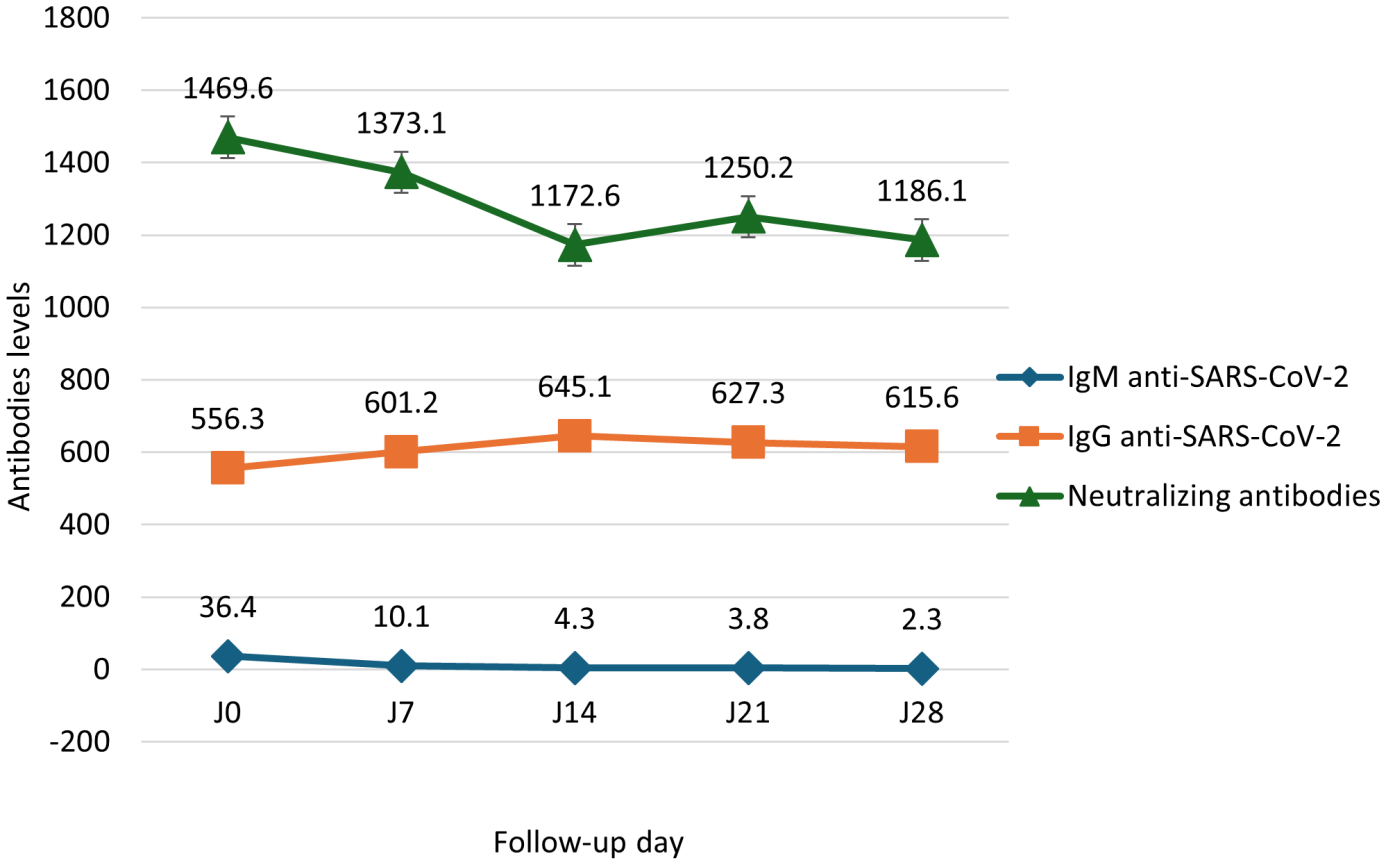
Median evolution of SARS-CoV-2–specific antibodies in positive cases (Day 0 to Day 28). Kinetics of SARS-CoV-2-specific antibody responses in COVID-19 patients. Levels of anti-SARS-CoV-2 IgM, IgG, and neutralizing antibodies were measured on days 0, 7, 14, 21, and 28 post-infection. IgM, IgG, and neutralizing antibody titers are expressed in Binding Antibody Units per milliliter (BAU/mL). Data are presented as medians with interquartile ranges (IQR); error bars represent the IQR.

**Figure 2:**
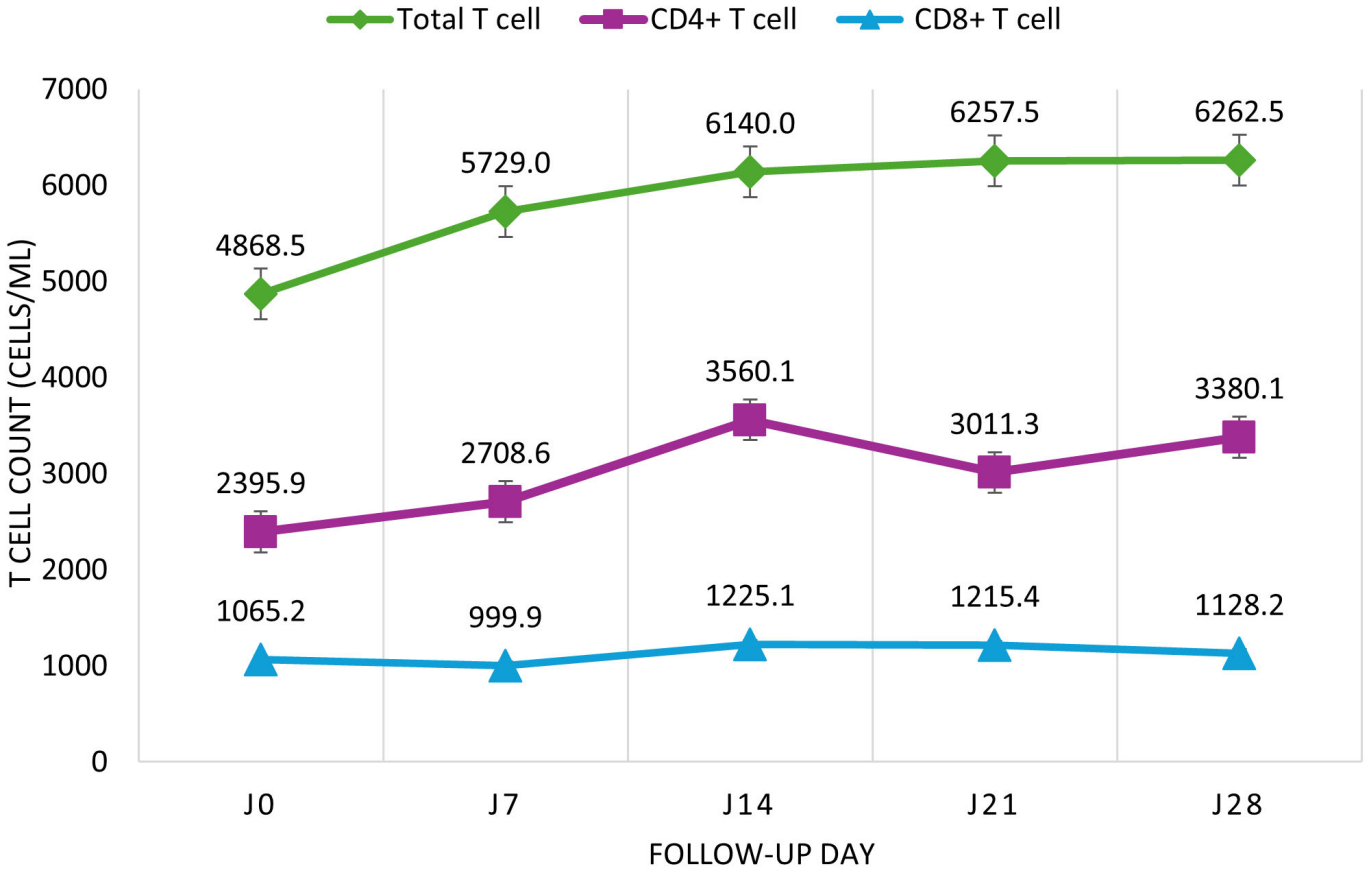
Median evolution of CD4^+^ and CD8^+^ T lymphocytes in COVID-19 cases from Day 0 to Day 28. Longitudinal evolution of T lymphocyte subpopulations in COVID-19 patients. Total T lymphocytes and CD4^+^/CD8^+^ subsets were quantified on days 0, 7, 14, 21, and 28 by flow cytometry. Cell counts are expressed in cells/µL. Data are presented as medians with interquartile ranges (IQR); error bars represent the IQR.

**Figure 3:**
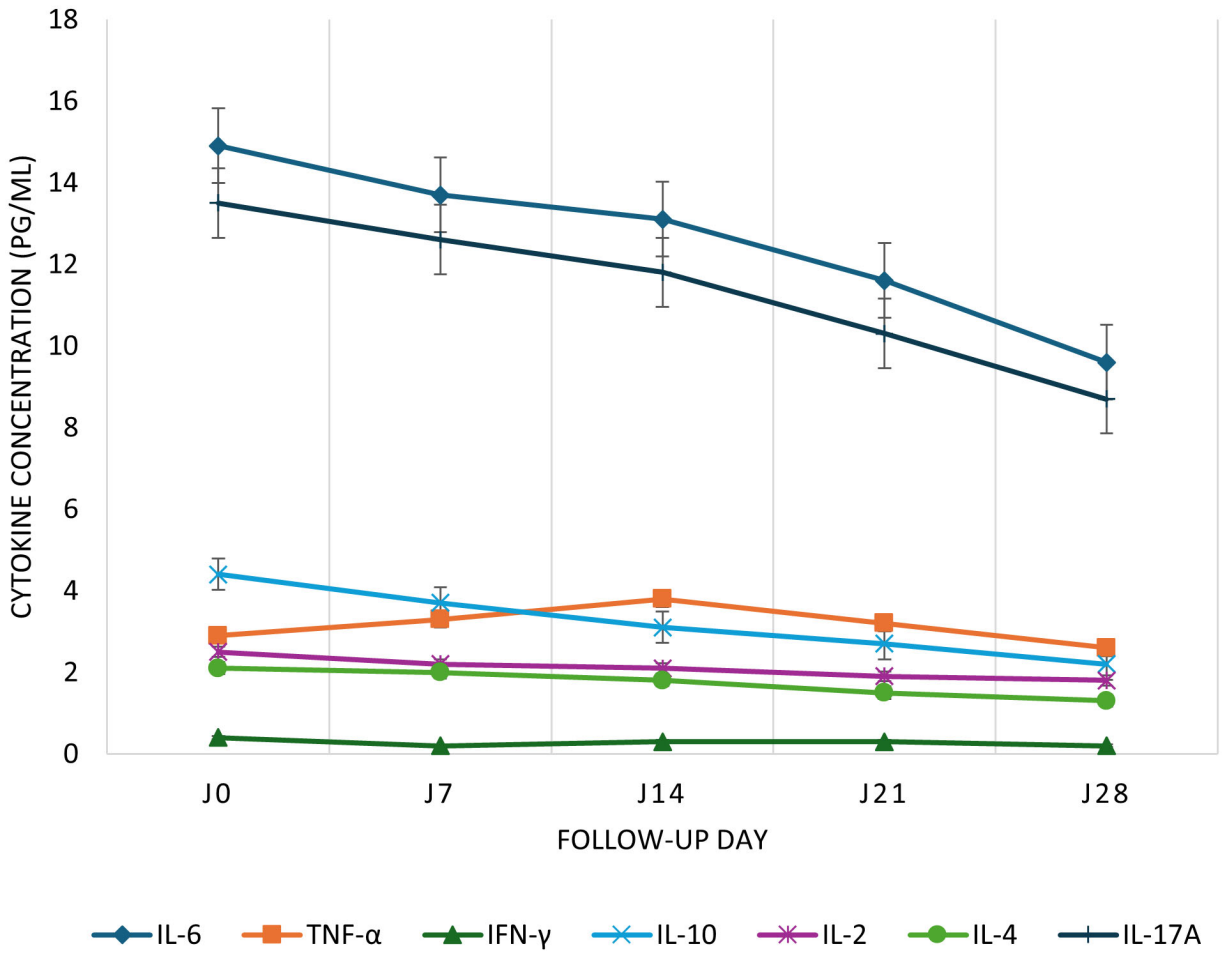
Kinetics of pro- and anti-inflammatory cytokines in COVID-19 cases from Day 0 to Day 28. Time course of plasma cytokine concentrations in SARS-CoV-2-infected patients. Plasma levels of IL-6, TNF-α, IFN-γ, IL-10, IL-2, IL-4, and IL-17A were measured at five time points during follow-up. Cytokine concentrations are expressed in pg/mL. Data are presented as medians with interquartile ranges (IQR); error bars represent the IQR.

IgM levels declined steadily from 36.4 to 2.3 BAU/mL between Day 0 and Day 28. IgG levels increased from 556.3 to 615.6 BAU/mL. Neutralizing antibodies decreased between Day 0 (1469.6 BAU/mL) and Day 14 (1172.6 BAU/mL), rose slightly at Day 21 (1250.2 BAU/mL), then dropped again at Day 28 (1186.1 BAU/mL; **Figure 1**). Total CD3^+^ T cells increased from 4868.5 to 6262.5 cells/μL. CD4^+^ T cells rose nonlinearly from 2395.9 to 3560.1 cells/μL between Day 0 and Day 14, dipped to 3011.3 at Day 21, and rose again to 3380.1 at Day 28. CD8^+^ T cells remained relatively stable between 999.9 and 1225.1 cells/μL (**Figure 2**). Pro-inflammatory cytokines such as IL-6 and TNF-α declined over time (IL-6: 14.9 to 9.6 pg/mL; TNF-α: 2.9 to 1.4 pg/mL), while regulatory cytokines IL-10, IL-2, and IL-4 showed modest increases from Day 7 to Day 28 (**Figure 3**).

## 4 Discussion

### 4.1 Humoral and neutralizing response

The observed humoral response is characterized by the production of specific IgG antibodies alongside neutralizing antibodies capable of preventing viral entry. In line with current knowledge, we observed a significant increase in antiviral IgG and detectable neutralizing activity from the second week post-infection (2, 14, 15). Neutralizing titers rose in parallel with IgG levels, peaking around week 4 (2, 14). This synchrony suggests that specific IgG directly contributes to viral neutralization, consistent with their documented role in viral clearance by blocking virus-cell binding (3, 4). We also demonstrated a positive correlation between IgG concentrations and neutralizing capacity, highlighting the importance of antibody quality in antiviral protection (4). This IgG-neutralizing antibody link has also been reported elsewhere, with both parameters rising in tandem about three weeks post-infection (14). Altogether, these findings confirm that high levels of specific IgG are closely associated with strong neutralizing activity, indicative of a robust and potentially protective humoral response. This includes some unvaccinated controls with IgG and neutralizing titers above threshold, likely due to undetected exposure or endemic coronavirus cross-reactivity.

### 4.2 Dominant CD4^+^ T Cell response and role of IL-2

The cellular arm of the adaptive immune response in our study is dominated by CD4^+^ T lymphocytes, with a more modest contribution from CD8^+^ T cells. CD4^+^ T cells were notable for their high IL-2 production, a cytokine essential for lymphocyte expansion and immune coordination. IL-2, mainly secreted by activated CD4^+^ helper T cells, plays a central role in promoting T and B cell proliferation and supporting NK cell activation (16). This T helper cytokine thus facilitates the coordinated rise of multi-compartment immunity. The observed strong correlation between activated CD4^+^ T cell frequency and IL-2 levels suggests a primarily Th1-type cellular response, focused on assisting other immune compartments. This Th1/IL-2-driven profile is frequently associated with effective immunity: in COVID-19, convalescent patients with favorable outcomes exhibit polyfunctional CD4^+^ T cells secreting IL-2, IFN-γ, and TNF-α (5, 11, 12), whereas severe cases often show loss of this functionality (17). Therefore, the predominance of IL-2-producing CD4^+^ T cells in our cohort may reflect a robust immune profile capable of supporting antibody production (via B cell help) and orchestrating the activation of other immune effectors.

### 4.3 Mixed cytokine profile (Th1/Th2/Th17)

Circulating cytokine measurements revealed a heterogeneous immune profile combining Th1, Th2, and Th17 features. In addition to canonical Th1 cytokines indicative of antiviral cellular responses (e.g., IFN-γ, IL-2, TNF-α), we simultaneously detected Th2-type cytokines (e.g., IL-4, IL-10) associated with humoral immunity and anti-inflammatory regulation, as well as the Th17 cytokine IL-17A. This combination suggests a non-polarized, multipronged T helper response. In SARS-CoV-2 infection, the virus has been reported to induce both proinflammatory Th1 and Th17 responses that may contribute to immunopathogenesis, while compensatory Th2 signaling may help mitigate excessive inflammation (18–21). The detection of IL-10 alongside IL-6 and TNF-α is consistent with previous reports highlighting the dual pro- and anti-inflammatory cytokine environment in moderate and severe COVID-19 (18) and reflects the regulatory role of IL-10 family cytokines in maintaining immune homeostasis (19). Our findings are consistent with such concurrent activation of multiple cytokine pathways. A mixed Th1/Th2/Th17 profile likely reflects the immune system’s effort to engage diverse arms of adaptive immunity to control infection while avoiding uncontrolled inflammation.

### 4.4 Specific role of IL-17A in asymptomatic individuals

A key finding of our study is the distinctive role of IL-17A in asymptomatic individuals. While IL-17A has been implicated in severe COVID-19 cases by fueling inflammation and cytokine storms (22, 23), our data suggest a different contribution in asymptomatic subjects. These individuals showed moderate but significant IL-17A production in the absence of clinical symptoms, implying a protective rather than pathogenic function. Recent studies have shown that some IL-17 family cytokines, such as epithelial-derived IL-17C, can locally boost barrier defenses and induce IL-17A expression to enhance innate immunity while limiting symptoms (24). Moreover, IL-17A, though identified as a marker of severe disease, is also induced during mild infections and remains elevated for up to 4 weeks post-infection (20, 24). It is therefore plausible that in asymptomatic individuals, a moderate and controlled Th17 response, marked by IL-17A, contributes to effective viral clearance (via neutrophil recruitment and mucosal barrier reinforcement) without causing tissue damage. This mechanistic interpretation aligns with the notion of infection tolerance seen in asymptomatic cases: these individuals may achieve viral control through efficient antiviral immunity while avoiding harmful inflammatory escalation (21, 24).

### 4.5 Immune kinetics over 28 days

Dynamic analysis of immune responses over 28 days revealed a well-coordinated sequence of phases. In the early days post-infection, innate immunity predominates, characterized by increased levels of inflammatory cytokines (e.g., IL-6 and TNF-α) in response to initial viral replication (1, 8). This acute phase is followed, from the second week onward, by the onset of specific adaptive immunity. Our data show that IgM and then IgG antibodies emerge during this period, with neutralizing activity becoming clearly detectable by 10–14 days post-infection (2, 14). Neutralizing titers, initially low in the first week, subsequently rise rapidly in parallel with specific IgG and typically peak around week 4 (2, 14, 15). Simultaneously, T cell responses emerge early: antigen-specific T cells can be detected by the end of the first week, with peak expansion occurring 1 to 2 weeks after infection (6, 25, 26). In our cohort, IL-2 and IFN-γ production by CD4^+^ T cells indicated strong cellular activation by the third week, suggesting that these cells reached maximal functionality at this stage. Finally, some cytokines such as IL-17A followed a later trajectory, with levels already elevated during the acute phase continuing to rise and remaining high through day 28 (24). This sequential pattern is consistent with longitudinal studies of COVID-19 describing a shift from early innate to robust adaptive immunity peaking between weeks 3 and 5 (2, 6, 25). In sum, our findings highlight an immune trajectory in which early innate responses prepare the ground, followed by humoral and cellular adaptive immunity reaching maximal strength by the end of the first month.

### 4.6 Cross-compartment correlations

Cross-analysis of immune compartments revealed significant correlations, underscoring coordinated orchestration of the anti-infective response. A strong correlation was observed between specific IgG levels and neutralizing activity: individuals with the highest IgG titers also showed the strongest neutralization capacity (4). This IgG-neutralizing antibody link confirms that humoral response quality (affinity and quantity of antibodies) largely determines neutralization efficacy. We also found a positive association between CD4^+^ T cell response magnitude and IL-2 production, consistent with the central role of helper T cells in IL-2 secretion to support cellular immunity (5, 16). In other words, stronger CD4^+^ T responses correspond to higher IL-2 availability, potentially amplifying effector lymphocyte expansion and antibody-secreting plasma cell generation. Furthermore, IL-6 and TNF-α levels were highly correlated, reflecting synchronous innate immune activation. These two mediators are often co-elevated in acute viral infections and act synergistically in inflammatory syndromes (1, 8). The observed IL-6-TNF correlation likely reflects shared upstream activation (e.g., via monocyte/macrophage pathways), a pattern frequently associated with more severe COVID-19 forms (27). Overall, these cross-compartment correlations emphasize that humoral, cellular, and cytokine responses do not operate in isolation but rather interact dynamically. Effective T cell responses facilitate antibody maturation (via CD4^+^ B cell help and IL-2 production), while innate inflammation modulates the context in which adaptive immunity unfolds. This multi-compartment coordination is key to a balanced and protective immune response.

### 4.7 Study limitations

This study has several limitations. First, the sample size was limited, which may reduce statistical power and the generalizability of findings. Second, the 28-day follow-up precluded assessment of longer-term immune durability, such as antibody titers beyond the first month or memory T cell persistence. Third, our analysis focused on selected immune compartments (IgG antibodies, CD4^+^ T cells, circulating cytokines) and did not assess potentially relevant actors such as cytotoxic CD8^+^ T cells, local innate immunity at infection sites, or mucosal IgA responses. These unmeasured elements could provide additional insights and merit future investigation. Finally, as an observational study centered on paucisymptomatic and asymptomatic cases, the reported correlations do not imply causality and may not be extrapolated to severe disease. Despite these limitations, our findings offer an integrated overview of the anti–SARS-CoV-2 immune response and pave the way for further investigations into mechanisms underpinning symptom-free infection and efficient viral resolution.

## 5 Conclusion

The immune responses observed in SARS-CoV-2-infected healthcare workers revealed a well-coordinated activation across humoral, cellular, and cytokine compartments. Sustained IgG and neutralizing antibody production, coupled with CD4^+^ T-cell expansion and cytokine modulation, reflected an adaptive response capable of controlling viral replication while minimizing inflammation. The immune profile of asymptomatic individuals suggests that effective viral clearance may occur through balanced, non-inflammatory mechanisms, possibly involving mucosal or Th17-mediated pathways. The detection of IgG and neutralizing antibodies in some uninfected controls likely reflects prior asymptomatic exposure or cross-reactive immunity. These findings provide valuable insights for improving immune surveillance and epidemic preparedness in African populations, while the limited cohort size underscores the need for larger longitudinal studies to confirm these observations.

## Data Availability

The original contributions presented in the study are included in the article/supplementary material. Further inquiries can be directed to the corresponding author.
